# Reproductive performance of Ankole cattle and its crossbreds in Rwanda

**DOI:** 10.1007/s11250-018-1658-8

**Published:** 2018-07-10

**Authors:** Maximillian Manzi, Lotta Rydhmer, Martin Ntawubizi, Callixte Karege, Erling Strandberg

**Affiliations:** 1 Swedish University of Agricultural Sciences (SLU), Uppsala, Sweden; 2 Rwanda Agricultural Board (RAB), Kigali, Rwanda; 3 University of Rwanda (UR), Butare, Rwanda

**Keywords:** Breeds groups, Fertility, Conception rate, Calving interval

## Abstract

The aim of this study was to assess the reproductive performance of Ankole cattle and its crossbreds with Friesian (F), Jersey (J), and Sahiwal (S). The traits (number of records) studied were calving to first insemination, CFI (797); calving to last insemination, CLI (797); conception rate, CR (4354); number of inseminations, NINS (936); and calving interval, CI (259). The overall means of intervals CFI, CLI and CI, CR, and NINS were 192, 198 and 480 days, 67%, and 1.23 respectively. Breed group was significant (*P* < 0.05) for all traits except NINS, while season of calving was significant for CFI, CLI, and CI, and season of insemination was significant for CR. The breed group AF had better CR than the purebred Ankole and AS, and AS had lower CR than AJxS and AJ. On the other hand, Ankole (and to some extent AF) had longer CFI and CLI than AJ, AS, and FF. Ankole had 54 days longer CI than all crossbreds taken together. The prolonged intervals CFI, CLI, and CI observed in this study call for proper postpartum anestrus management both in terms of nutrition and calf suckling management.

## Introduction

Dairying in Sub-Saharan Africa based on indigenous breeds is not a suitable option to meet the increasing demand of milk and milk products because of their low production potential compared to breeds selected for high production. Despite the scientific criticism of indiscriminate crossbreeding, many farmers in favorable environment with market opportunities in place often go for this option that combines the hardiness of *Bos indicus* with the production capacity of *Bos Taurus* animals (Wurzinger et al. 2014). For many regions of Africa, there is a need for research looking into sustainable crossbreeding strategies and to determine best composites, both in terms of breeds involved and the proportion of each breed (Wurzinger et al. 2014). The reproductive performance of dairy cows is the most important prerequisite for sustainable dairy production systems (Nuraddis and Ahmed 2017). Reproductive performance is not only affected by herd-level management factors (such as poor nutrition, incorrect estrus detection, semen handling, and cow management) but also cow-level factors (such as prolonged postpartum anestrus, calving interval, interval to first service, and ovarian disorders) (Hudson 2011).

In Rwanda, crossbreeding has been part of the government policy, in efforts to fast-track increase in milk and meat production. This has been achieved through state-subsidized artificial insemination (AI) campaigns, in which hundreds of thousands of cows have been bred. Synchronization of estrus has also been partly instrumental in the process. Nevertheless, there is limited or no information on the reproductive performance of Ankole cattle and its crossbreds and factors that influence these performance traits under prevailing local conditions. With the long-term goal to improve reproductive performance of dairy cows in Rwanda, the aim of this study was to assess the reproductive performance of Ankole cattle and its crossbreds using performance records from three research stations in Rwanda.

## Material and methods

### Location

Information was obtained from reproductive records from experimental stations at Songa (2° 24′ S, 29° 46′ E), Nyagatare (30° 25′ E, 2° 30′ S), and Kinigi (01° 43′ S, 29° 54′ E), located in altitude 1600, 1425, and 2400 m.a.s.l. respectively. The rainfall pattern is bimodal, with short rains (season SRS) falling between September and December and long rains (season LRS) extending from March through May. The dry seasons extend from June to August (LDS) and January to February (SDS). The rain is heavy in March and April, and decreases gradually in May. The mean annual rain fall and temperature for the three station were 1087 mm and 20.1 °C, 850 mm and 25.3 °C, and 1650 mm and 16 °C respectively during the years 1998–2017.

### Management of animals

The animals were raised entirely on natural pastures without supplementary feeding, except mineral licks given ad libitum. Only younger calves, cows calving, and sick cows were housed, while others were left freely to move in their paddocks all day round. Water was provided twice daily. Culling was based on subjective opinion of old age (e.g., loss of teeth) and infertility. Routine disease control measures were undertaken and these included treatments of animals against ectoparasites and endoparasites. The animals were vaccinated when the need arose, especially against FMD and routinely against Anthrax and CBPP and Blackleg. Lactating cows were normally returned in the barn where they were milked.

All year round, breeding was practiced and heat detection was based on observations and reports from trained herdsmen. Animals noted in heat in the morning were inseminated that afternoon and those identified in the afternoon were inseminated next morning based on the “am-pm guideline” (Peter and Ball 1995).

### Data collection

Reproductive performance records were from purebred Ankole (AA) and crossbreds with Friesian (F), Jersey (J), and Sahiwal (S). Breed groups (crosses) are designated by the combination of breed acronyms, e.g., AJxS for a cow with an Ankole x Jersey crossbred mother with a Sahiwal father. The available breed groups with sufficient number of cows were AA, AF, AJ, AJxS, AS, and ASxJ.

Insemination was done by 14 different professional AI technicians at variable times after the first signs of estrus (from 6 to 35 h after). The following data were recorded for each animal: breed group, date and time an animal was observed in heat, estrus type (induced estrus by PGF_2**α**_ or natural estrus), date and time the cow was inseminated, service sire, and AI technician. Conception rate (CR) was based on the success or failure of individual first inseminations. If an insemination was not followed by another insemination within 270 days, it was considered as resulting in a pregnancy (designated by 1); otherwise, it was considered a failure (0). Calving interval (CI) was estimated as difference in days between two successive calvings. Calving to first service interval (CFI) and calving to last insemination (CLI) were calculated as days elapsed between calving and first insemination or last insemination, respectively. Number of inseminations (NINS) per series was calculated by defining a series when inseminations were within 56 days of each other. The dependent variables considered were CR, CFI, CLI, NINS, and CI. Lower (upper) limits (inclusive) for dependent variables were set to CFI and CLI 30 (375), NINS 1 (4), and CI 300 (750).

Factors studied affecting these traits were the effect of breed group, season of AI (for CR) or calving (for CFI, CC, and CI) (four season classes SDS, LRS, LDS, SRS), and year of insemination (1999, …, 2010, 2013, …, 2017) or year of calving (1999, …,2004, 2014, …, 2017; for some traits with very few observations in a year, that year was combined with the following year), and station. Because there were very few (or no) crossbreds in the early years, we also combined calving years into a broader time period (up to 2004 and 2014 and later), to avoid confounding of year and breed. For CR, insemination bull breed (J, S, or H), AI technician (14 levels), and time elapsed from detection of estrus to insemination (4–8, 9–12, 13–16, 17–20, 21–24, and > 25 h) were also considered. The assignation of parity was uncertain, what is designated as first parity was actually first *known* parity, which may or may not have been the actual first parity. We only distinguished between first known parity and any later parities. Factors were considered statistically significant at a *P* value < 0.05.

### Statistical analysis

The statistical analysis was carried out using the SAS software (2012). The effects that were found to be significant were included in the final model for each trait. Conception rate (CR) was analyzed using a logistic regression in Proc GLMMIX with a logit link function, whereas for CFI, CLI, NINS, and CI, a fixed linear model using Proc GLM was used.

## Results

### Intervals from calving to first or last insemination

The average CFI was 192 days (*n* = 797, SD = 87) and the average CLI was only slightly longer 198 days (*n* = 797, SD = 88). Breed group (AA, AF, AJ, AS, FF) and season had significant effect on both traits, whereas time period and station did not. Purebred Ankole had longer CFI and CLI than all other breed groups, except for AF; the latter had longer intervals than FF (Table 1). The estimate for AF was based on very few animals and might therefore not be representative for that cross.

**Table 1:** Number of observations and least square (marginal) means (±SE) for breed groups and seasons from the linear models or logistic regression (for CR) for intervals from calving to first or last insemination (CFI, CLI, both having the same number of observations), calving interval (CI), and conception rate (CR)

Variable^1^	Traits^2^						
	*n*	CFI	CLI	*n*	CI	*n*	CR^3^
Breed group							
AA	636	202 ± 3.4^a^	208 ± 3.5^a^			2480	0.71 ± 0.016^bc^
AF	14	204 ± 23^ab^	207 ± 24^ab^			282	0.78 ± 0.026^a^
AJ	60	170 ± 11^b^	176 ± 11^b^			585	0.73 ± 0.021^ab^
AJxS						221	0.75 ± 0.031^ab^
AS	60	156 ± 11^bc^	163 ± 11^bc^			567	0.67 ± 0.024^c^
ASxJ		–	–			219	0.71 ± 0.034^bc^
FF	19	120 ± 19^c^	131 ± 19^c^				
Season							
SDS 3	127	189 ± 8.4^b^	197 ± 9.6^a^	48	486 ± 14^a^		
LRS 2	237	172 ± 8.2^ab^	179 ± 8.4^ab^	84	462 ± 11^ab^		
LDS 4	200	190 ± 9.4^a^	170 ± 8.8^bc^	62	442 ± 13^b^		
SRS 1	225	157 ± 8.4^a^	163 ± 8^c^	65	495 ± 12^a^		

^1^AA = Pure Ankole, AF = Ankole x Holstein Friesian, AJ = Ankole x Jersey, AS = Ankole x Sahiwal, AJxS = AJ x Sahiwal, ASxJ = AS x Jersey, FF = Pure Holstein Friesian, SDS = short dry season (Jan–Feb), LRS = long rainy season (Mar–May), LDS = long dry season (Jun–Aug), SRS = short rainy season (Sep–Dec)

^2^*n* = number of observations, CFI = calving to first insemination, CLI = calving to last insemination, CI = calving interval, CR = conception rate

^3^Values transformed back to the original scale

^abc^Mean values within breed group or season with different letters are significantly different (*P* < 0.05)

### Conception rate

The overall mean CR was 0.67 (*n* = 4354, SD = 0.47). The factors’ breed group (AA, AF, AJ, AJxS, AS, ASxJ), year of insemination, and technician were significant in the logistic model. There were too few FF to be included in the analysis. The genotype AF had better CR than the purebred Ankole and AS, and AS had lower CR than AJxS and AJ (Table 1).

**Fig. 1:**
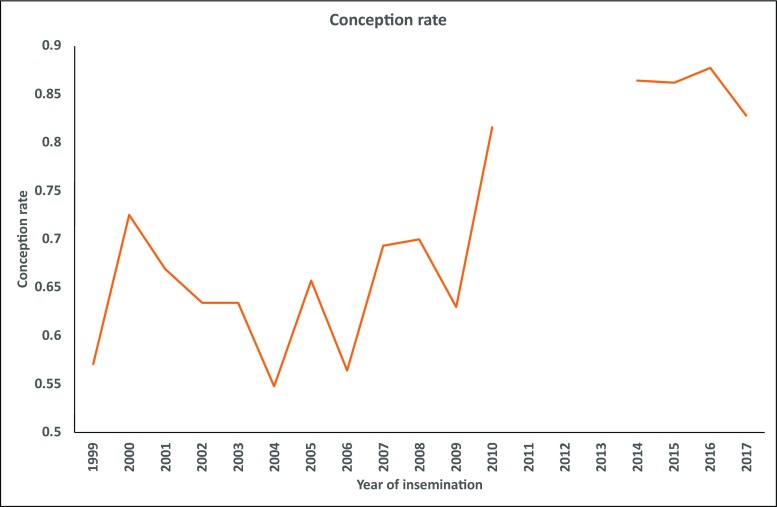
Conception rate estimates by year of insemination from logistic regression model. Note that there are some years with missing information

Changes in CR over time were observed. There was a tendency for an increasing CR over time; however, there were substantial fluctuations between adjacent years (Fig. 1).

Significant variation was observed in results among technicians. The difference between the lowest and highest technician was more than 20 percentage units.

Despite an almost 5 percentage units lower CR for induced vs non-induced estrus, the results were not significant (*P* = 0.07). However, the interaction between breed group and estrus type was significant (*P* < 0.01). Some breed groups followed the overall tendency of lower CR for induced estrus, whereas others had the opposite results (AJxS and ASxJ) (results not shown). For AA, there was no difference; the only significant difference was for AS, where induced estrus had much lower CR (0.49) than non-induced estrus (0.68).

### Number of insemination per series

The overall mean value of NINS was 1.23 (*n* = 936, SD = 0.54). Only time period was significant for NINS. The first period (up to 2004) had slightly higher NINS than the period from 2014 onwards (1.32 vs 1.18), which is consistent with the time trend for CR (Fig. 1).

### Calving interval

In total, there were 186 first parity cows with information on CI and 73 from second or later parities. Owing to the few number of records, it was not possible to have the same definition of breed groups. Instead, all crosses were grouped together and compared with purebred Ankole. After first fitting breed group (AA or cross), which was significant, season was significant but not time period, parity, or station.

The overall mean calving interval observed was 479 days (*n* = 259, SD = 103). Ankole had about 54 days longer CI than the crossbreds (LSM 498 (SE 7.7) vs 445 (SE 10.8).

The season of calving had significant effect on CI. Significantly lower CI were observed in LDS (442 days) compared to SDS (486 days) and SRS (495 days); however, SDS and SRS did not differ among each other (Table 1).

## Discussion

### Intervals from calving to first or last insemination

The trait calving to first or last service plays an important role in reproductive efficiency in dairy cattle. The average CFI (192 days) and CLI (198 days) obtained in this study were higher than CFI of 159 days and CLI of 178 days for Sanga and CFI of 115 days and CLI of 138 days for Sanga x Friesian reported in Ghana (Obese et al. 2009). In a review by Nuraddis and Ahmed (2017), lower, comparable, and higher calving to conception values (120–316 days) were reported for crossbreeds of Holstein x Zebu, Friesian x Horo, and Friesian x Arsi in Ethiopia, and in the same review, the CFI of 165 days for Friesian x Zebu was also reported. The CFI values (120–204) recorded in this study (Table 1) were longer than the recommended 45–60 days considered optimum to achieve annual calf crop target. The reasons for the delay could be due to postpartum anestrus as result of poor nutrition and suckling management (Diskin et al. 2003, Robinson et al. 2006). Earlier studies in the Songa herd indicated that there was lack of restriction on suckling of calves until they were weaned subjectively between the ages of 8 to 13 months (Manzi et al. 2018). Prolonged suckling stimulus could delay resumption of ovarian cyclicity by interfering with hormones responsible for follicular development (Thatcher et al. 2006). Therefore, better management practices including early weaning and restricted suckling could overcome this deficiency by shortening number of days opens, leading to reduced calving intervals.

### Conception rate

The CR in this study was affected by breed group, year of insemination, and AI technician, while the contribution of station, service sire, insemination season, and time of insemination to the total variance in the CR was minor. The CR in our results ranged from 67 to 78% for different breed groups (Table 1). Tahmina et al. (2016) reported comparable CR (76%) for the crosses of Holstein Friesian and local breeds in Bangladesh, while in the same country, Khan et al. (2015) reported conception rates of 64% in native cattle, 57% in Friesian cross, and 53% in Sahiwal cross. In Ethiopia, Debir et al. (2016) in a study of AI efficiency reported conception rates in indigenous (54%, *n* = 98) and crossbred cows (70% *n* = 69), while Woldu et al. (2011) in an on-farm study reported conception rates of 33% (*n* = 58) in indigenous cows and 59% (*n* = 87) in crossbred cows.

Some authors, as in our study, have reported inter-technician variability in CR. For example, Siddiqui et al. (2013) reported significant effect of technician on the conception rate in smallholder farms in Bangladesh with the difference between the highest (58.6%, *n* = 512) and the lowest (43.4%, *n* = 281) being 15.2%. Also, Miah et al. (2004) in the same country reported significant effect of AI technician on conception rate that varied based on experience, with average conception rates of 56, 67, and 68% in cows inseminated by technicians with 1–2, 2–3, and 3–5 years of experience, respectively. Even though no recent publications on the reasons for differences between AI technicians exist, in New Zealand, Visser et al. (1988) reported 19% of the explained variation in conception rate to be attributed to the individual AI technician. Barth (1993) in a study on factors affecting fertility with AI in North America cited personal qualities or personal problems as possible reasons for differences in performance between individual technicians.

The trend in CR over time (Fig. 1) showed an increasing tendency; this might be attributed to gradual improvement in herd management, and with continuous guidance and training, the inseminators acquired more skills with time.

### Number of inseminations per series

Number of services is one of the most important factors that directly affects fertility results of the herd. All factors except time period (1999–2004 vs 2014–2017) were non-significant. In Ethiopia, Tesfa and Garikipati (2014) reported significant effect of the year but non-significant effect of genotype in a review study on the reproductive performance of crossbreeds and their respective indigenous breeds. In a review by Nuraddis and Ahmed (2017), several authors reported number of services per conception to be dependent largely on the breeding system use (higher under controlled natural breeding than hand mating and artificial insemination) and that values of greater than 2 should be considered as poor. Therefore, in this study, the NINS mean values obtained in the two time periods, 1.23 for 1999–2004 and 1.18 for 2014–2017, were below recommended value and the difference in time periods could be due to improvement in herd management and insemination practices over time.

### Calving interval

The length of postpartum anestrus and service periods are part of the calving interval that can be shortened by improved herd management. To achieve a calving interval of 365 days, “open days” should not exceed 80–85 days, which is optimal to obtain annual calf crops (Nuraddis and Ahmed 2017). The mean CI (480 days) obtained in the present study is not within the range (365–420) considered optimal for tropical cattle breeds (Aboagye 2002). The extended CI seems mainly to be the result of prolonged interval from calving to first service. In Mexican tropical environment, Segura-correa et al. (2017) reported long CI of 446 and 481 days for Brahman and Guzerat, respectively, and attributed this poor performance to tropical conditions that limit the reproductive functions of cows such as heat stress and poor-quality pasture. Vinothraj et al. (2016) reported CI of 489 days for crossbred Jersey and Red Sindhi in India, while in Ethiopia, Nuraddis and Ahmed (2017) reported even longer CI (630 days) for crosses of Friesian and Zebu. In Ghana, Samuel and Julius (2014) recorded shorter CI (413 days) for Sanga and crossbreds of Sanga with Friesian.

The seasonal influence on CI (Table.1) obtained in this study is in agreement with the report by Fekadu et al. (2011) who attributed the difference in CI with season to be due to cows calving in dry season taking advantage of the improved feed availability in the subsequent rainy season, enabling the animals to meet their total needs for maintenance, lactation, and resumption of estrus cycle (Ruegg 2001).

### Concluding remarks

The conception rate showed an improving tendency across years and was within acceptable levels, an indication of gradual improvement in the herd performance. Although compared to other breed groups, purebred Ankole had longer CFI, CLI, and CI; intervals for these traits were longer than desirable for all breed groups, which is unfavorable for a profitable cattle production. Therefore, strategies aimed at improving the reproductive performance should be adopted. These should include early weaning, restricted suckling, and proper feeding which aid in shortening of postpartum anestrus. Also, these extended intervals may be indicators of missed estrus signs and/or poor record keeping. Therefore, more focused studies are recommended to fully realize the genetic potential of these animals.
